# An Overview of Guidelines for Supplemental Feeding of Infants in Swedish Maternity Clinics

**DOI:** 10.3390/nursrep11010010

**Published:** 2021-02-07

**Authors:** Birgitta Kerstis, Anna Richardsson, Alexandra Stenström, Margareta Widarsson

**Affiliations:** 1 School of Health, Care and Social Welfare Mälardalen University, S-722 18 Västerås, Sweden; margareta.widarsson@mdh.se; 2 Department of Obstetrics and Gynecology, Örebro University Hospital, S-701 85 Örebro, Sweden; Anna.richardsson@regionorebrolan.se (A.R.); Alexandra.stenstrom@regionorebrolan.se (A.S.)

**Keywords:** content analysis, guidelines, infants, maternity ward, supplemental feeding, Sweden

## Abstract

This study aims to describe the local guidelines for the supplemental feeding of infants of Swedish women’s clinics with maternity wards. Purposeful sampling was used during a four-week data collection time in 2019. Guidelines from 41 of the 43 Swedish women’s clinics with maternity wards were analysed using qualitative and quantitative content analysis. The information provided, and length of the guidelines varied widely in 38 guidelines. Feeding methods were included in 28 guidelines, but 10 provided no information about feeding methods. The most common feeding methods were cup feeding and feeding probes. Suggestions for supplemental feeding included infant formula (32), breast milk (27) and no suggestions (6). The methods to support breastfeeding were skin-to-skin contact (25), breastfeeding freely (22), a caring plan (18), extra supervision (3), optimising the caring environment (2), supplying a breast pump (1) and breastfeeding observation (1). Twenty-two guidelines included information about how long formula should be given and that the feeding should be phased out gradually. We conclude that a national guideline for the supplemental feeding of infants is needed to ensure equal best practice care for infant safety and the support of parents to increase the breastfeeding rate. More national guidelines are needed in general because it is easier to update only one set of guidelines.

## 1. Introduction

The World Health Organisation (WHO) recommends exclusive breastfeeding for 6 months and part breastfeeding for up to 2 years of age. In 1991, the WHO and the United Nations Children’s Fund created the Baby-Friendly Hospital Initiative, which includes the global strategy Ten Steps to Successful Breastfeeding [1]. This strategy aims to protect, promote and support breastfeeding and to enhance the conditions for successful breastfeeding worldwide. In Sweden, a guideline for hypoglycaemia in neonates [2] focuses on the medical aspects, but there is no national guideline for use in maternity wards on the supplemental feeding of infants. This lack of guidance may cause inequalities in the care of infants and the advice given to parents and may prevent the delivery of best practice and evidence-based care. Providing this knowledge may help to provide better support for parents about breastfeeding and supplemental feeding.

The Swedish National Board of Health and Welfare defines complete breastfeeding as giving only breast milk, vitamins and any medication, and part breastfeeding as additional formula breastfeeding [3]. In the 1930s, the number of hospitalised deliveries in Sweden increased and children were often nursed in separate rooms and at scheduled times. The separation of child and mother continued for many decades and reduced the opportunities for skin-to-skin contact, which promotes breastfeeding and milk production [4,5,6]. During the 1950s and 1960s, breast milk replacement was marketed as being equivalent to breast milk and helping to free women from children and the home [7]. At the beginning of the 1970s in Sweden, about 5–7% fully breastfed at six months. Positive attitudes about breastfeeding and the health effects of breastfeeding increased the number of breastfed infants during the 1990s [8]. The percentage of children fully breastfed at 6 months has remained unchanged at 13% since 2010 [3]. According to Rollins et al. (2016), health systems and community interventions can increase the rate of exclusive breastfeeding by 2–5 times [9]. Therefore, guidelines in accordance with available evidence and with evidence-based recommendations are necessary to achieve the adequate management of supplementary feeding for infants. National guidelines will support standardised regimes [10].

The nutritional characteristics of breast milk vary between individuals [11], and breastfeeding can decrease the frequency of bacterial infection, ear inflammation, obesity and sudden infant death [11,12,13]. Breastfeeding can also reduce the risk of overweight and obesity throughout the child’s life [12,13], and can positively affect cognitive development [14,15]. Breastfeeding also has positive health effects on women by reducing the risk of breast and ovarian cancer [13,16,17].

After childbirth, the child needs to produce glucose until the mother’s breast milk production starts, and a healthy full-grown neonate can handle this adjustment without problems [2]. The risk factors for hypoglycaemia are premature birth, low birth weight, large for gestational age (LGA), perinatal asphyxia, respiratory disorder, infection, hypothermia, twin delivery and/or maternal diabetes [18,19]. Strategies to prevent hypoglycaemia in infants include skin-to-skin contact, blood glucose monitoring until achieving two recordings >2.4 mmol/l and encouraging early frequent breastfeeding [20]. Supplemental feeding is another way of preventing hypoglycaemia [10]. The risk factors for hypoglycaemia are maternal diabetes and early gestational age [18]. Untreated hypoglycaemia can cause poor feeding, hypotonia, hypothermia, jitteriness, tremulousness and irritability [19,21]. Persistent hypoglycaemia can cause respiratory distress, seizures and brain injury in infants [19,22]. In high-risk children, it is important to breastfeed and give supplements to avoid persistent low blood glucose levels [19,23]. Therefore, it is important to provide supplemental feeding to infants who are unable to wait until the mother’s milk flows, or infants who are unable to suck for any reason. Feeding guidelines are intended to prevent low blood sugar levels, which could cause serious consequences [24]. In Sweden, a shortage of donated human milk sometimes exists, and therefore neonatal clinics should prioritise the premature and/or sick infants rather than healthy infants at maternity clinics. Furthermore, Swedish mothers are only allowed to donate breastmilk for three months after delivery [24]. Supplementary feeding can be performed by cup, bottle and feeding tube with breastmilk or infant formula [25].

A Spanish study reported that 84% of women wanted to breastfeed before delivery but, when they left the hospital, only 58% fully breastfed [26]. A metanalysis found that 37% of children younger than 6 months were breastfeed exclusively in low-income or upper-middle-income countries; by contrast, the rates were reported as 20% in high-income countries and 16% in Sweden [13]. A Canadian study emphasised the importance of fathers in the decision-making regarding how their infants are fed [27]. Possible ways to reduce formula feeding at hospitals are prenatal education, peer counselling, hospital staff and physician education, and skin-to-skin contact [28].

According to a clinical protocol from the United States, these are widespread recommendations concerning supplemental feeding rather than national guidelines [29]. Previous research has not examined local guidelines for the supplemental feeding of infants in Sweden. What is known is that breastfeeding can improve both the child’s and mother’s health. The National Board of Health and Welfare in Sweden has regulations that provide binding rules stating that breast milk compensation should only be given to infants for medical reasons [30]. There is a critical need to investigate the local guidelines for supplemental feeding to aid decisions about national guidelines to strengthen patient safety and ensure that the requirement for equal care is met. This knowledge will help health professionals better identify problems and support parents with breastfeeding, which could facilitate best practice care for children. Therefore, the aim of the present study was to describe the local guidelines for the supplemental feeding of infants used in Swedish women’s clinics with maternity wards.

## 2. Material and Methods

### 2.1. Collection of Data

A purposeful sampling was used in 2019, when all 43 of the Swedish women’s clinics with maternity wards were requested for their guidelines regarding the supplemental feeding of infants. There are no privately run maternity clinics in Sweden caring for infants that are in need of supplemental feeding.

Each hospital switchboard was contacted to provide addresses and phone numbers for the unit heads of the delivery wards, information that is not classified. Subsequently, an information letter was mailed to that person describing the study’s aim, methods and information about voluntary participation. Of the 43 women’s clinics contacted, 41 responded (95%) during the 4-week data collection. Three of the responding clinics had no current guidelines, and a total of 38 clinics were included in this study.

### 2.2. Data Analysis

The study’s design involved content analysis according to Elo and Kyngäs (2008), which is useful when the aim is to describe a phenomenon [31]. The deductive approach is appropriate when the material already exists but is to be examined in a new context [31]. We also performed quantitative content analysis and reported the number of guidelines in each category, which added valuable information about differences in content between guidelines.

The guidelines were read and compared by checking the accuracy of the text and identifying contents related to the study’s aim. In the next step, similarities and differences were identified. The authors read each guideline independently and highlighted text that was relevant to the research question. In the next step, each guideline’s content was analysed and sorted into nine categories (Table 1) which was used as a protocol for the content analysis. A quantitative content analysis was also performed to describe the frequency of content areas. The data were then compiled and are presented in the results section in the form of text, tables and figures. Throughout the process, the research group worked together to achieve consensus in the analysis. The GRADE (Grading of Recommendations, Assessment, Development and Evolution) approach was used as a standardised judgement tool [32]. This study was approved by the local university board and was conducted in accordance with the Declaration of Helsinki [33].

## 3. Results

Descriptive data obtained from the 38 guidelines are presented in Table 2. Eighteen referred to The National Care Program–Neonatal hypoglycaemia in infants with gestational age ≥ 35 weeks, which had a high to low level of evidence GRADE (Table 3). Five of the 38 guidelines also included other references, and 19 did not. Of those, four had a moderate and one a low level of evidence GRADE [32].

There were no differences between the nine categories, whether the guidelines stemmed from the capital or from a smaller area.

### 3.1. Feeding Methods and Risk Factors

**Figure 1:**
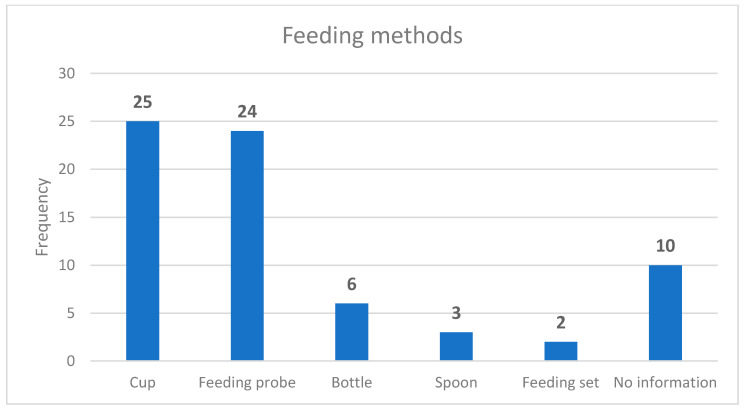
Feeding methods for supplemental feeding.

Feeding methods were included in 28 guidelines, and 10 provided no information about feeding methods (Figure 1). The most common feeding methods were cup feeding and feeding probe. Supplemental feeding was suggested mainly as infant formula (*n* = 32) and breastmilk (*n* = 27); six guidelines made no suggestions.

**Figure 2:**
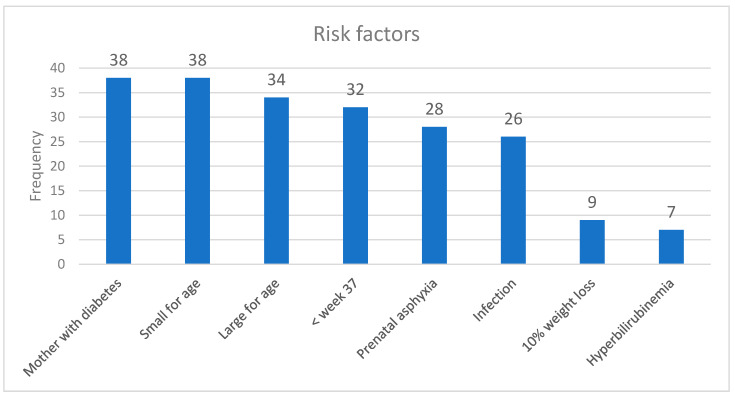
Risk factors for the need for supplemental feeding.

The most common risk factors for supplemental feeding were maternal diabetes, small for gestational age, LGA, born before week 37, perinatal asphyxia, infection, 10% weight loss and hyperbilirubinaemia (Figure 2). The guidelines included mothers with diabetes type 1, diabetes type 2, and diabetes in pregnancy in combination.

### 3.2. Breastfeeding Support

The guidelines included ways to support breastfeeding such as skin-to-skin contact (*n* = 25), breastfeeding freely (*n* = 22), a caring plan (*n* = 18), extra supervision (*n* = 3), optimisation of the caring environment (*n* = 2), provision of a breast pump (*n* = 1) and breastfeeding observation (*n* = 1). Half of the guidelines stated that breastfeeding should be initiated within 1 h after birth and advocated ad lib breastfeeding between supplemental feeding. Twenty guidelines stated that a mother of a supplemental-feeding child should receive instructions about using a breast pump to keep their milk production going. Twenty-four guidelines emphasised that supplemental feeding should be used only under strict medical supervision because of the risk of disturbed breastfeeding. Two guidelines described the importance of supporting mothers’ breastfeeding by not making her feel inadequate by using supplemental feeding. Three guidelines mention some sort of support to encourage breast feeding and to yield her milk by hand or pump.

### 3.3. Discontinuation of Supplemental Feeding

Twenty-two guidelines included information about the duration and gradual phasing out of supplemental feeding. One described that, before discharge, an individual assessment should be made after breastfeeding observation. Five recommended visits to the maternity clinic after discharge for a follow-up.

## 4. Discussion

To our knowledge, this is the first study to describe guidelines for the supplemental feeding of infants in Sweden. We received guidelines from 41 of Sweden’s 43 women’s clinics with associated maternity wards. We reviewed the guidelines from 38 clinics, because two had not updated their guidelines and one had none. Most guidelines described similar risk factors for requiring supplemental feeding. Many also included information about the advantages of breastfeeding.

The main findings of our analysis revealed much variance in supplemental feeding management between clinics. Most guidelines included maternal diabetes as a risk factor, which is in accordance with other studies [18,19]. The type of maternal diabetes was seldom mentioned, which is noteworthy because this is important when deciding on the planned interventions. It is important to facilitate skin-to-skin contact for all infants, especially for children whose mother has diabetes, because this can prevent hypoglycaemia, and to encourage early frequent breastfeeding [20]. Early breastfeeding stimulates the breastmilk, and it is important to promote skin-to-skin contact and sucking, in both full-term and premature babies [6].

The national guidelines for neonatal hypoglycaemia, used by 18 clinics in our study [2], may provide the basis for creating a national guideline for supplemental feeding. However, these should be expanded to include caring aspects such as parental support and skin-to skin contact. The advantages of breastfeeding for both infant and mother are well known [13,22]. Despite this, infant formula is suggested by the current guidelines’ as the main form of supplemental feeding. Further, infants receiving infant formula during the first week of life stop breastfeeding earlier than those who fully breastfed from birth, even if the mother’s intention is to breastfeed fully [26,34].

One dilemma is that unnecessary supplemental feeding can decrease the use of breastfeeding [28] which can limit the positive health effects for both infants and mothers [13]. However, it is important to have clear medical indications for supplemental feeding to prevent exposing children to the risk of hypoglycaemia. We suggest that mothers are encouraged to breast feed if possible, even if supplemental feeding is performed when the infant’s blood sugar level is low. Hypoglycaemia can cause severe symptoms such as weak suction, hypothermia, sweating, shaking, irritability, drowsiness, decreased tone, seizures, apnoea, tachypnoea, bradycardia and/or circulatory arrest and in extreme cases, brain damage [22,35]. We emphasise that guidelines for the supplemental feeding of infants need to focus more on the caring perspective, because this can help ensure health professionals better support mothers who are breastfeeding. Caring perspectives can include prenatal education, skin-to-skin contact, peer counselling, hospital staff and physician education [24,28].

It is disadvantageous that only 28 of the guidelines examined describe how infants should receive supplemental feeding, and that 25 guidelines recommended cup feeding, and 24, the use of a feeding probe. This is in accordance to Flint’s (2017), description of how infants should receive supplemental feeding [25]. Thirty guidelines mentioned breast milk and breast milk formula for supplemental feeding. When possible, the mother’s own breast milk should be used [2,36]. If mother–infant separation in unavoidable, the mother needs instruction, practical help, encouragement, and support from professionals to yield her milk by hand or pump to stimulate milk production [24,29]. To avoid giving formula supplementation to healthy infants, the medical indications should be clarified for both parents and health professionals, and hospital policies should be updated [37]. This reinforces the importance of developing common guidelines for the whole country. Breastfeeding contributes to better health, and it has been suggested that breastfeeding can be improved with interventions, programs and policies that promote, protect and support breastfeeding [9].

Several guidelines included promoting skin-to-skin contact immediately after birth and breastfeeding freely. These also focused on initiating breastfeeding early and advocated breastfeeding between feeding times. In addition, 22 guidelines mentioned ways to continue milk production and how supplemental feeding should be discontinued. WHO states that breastfeeding support should be promoted to facilitate breastfeeding; one example of such promotion is to avoid using a pacifier in the first 2 weeks of life [1]. However, studies have found no negative association between breastfeeding and early versus late introduction of a pacifier [38,39]. This contrasts with the findings of Batista et al. (2019), who suggested that the use of artificial nipples can be associated with changes in the sucking patterns of infants, which can have negative effects on breastfeeding [40]. It is important to have overall national best practice guidelines rather than each facility using its own. This will help to ensure constancy in the quality of care for infants and mothers.

Parental support was rarely described in the guidelines, which focused more on medical issues relating to the child. We emphasise the importance of parents feeling supported and the involvement of both parents throughout the pregnancy, childbirth and postpartum. This is consistent with other studies showing that the provision of professional support to expectant couples increases the partner’s ability to communicate and to experience togetherness [41,42]. Support for and information on breastfeeding should include both parents and be delivered using a variety of modes [43]. More attention should be given to the father’s role in breastfeeding because he is important in the decision making [27].

Women who experience complications during childbirth, such as caesarean delivery or foetal or postpartum haemorrhage, are more likely to breastfeed for a shorter time than women who do not experience complications [39]. Caesarean-delivered infants need more supplemental feeding compared with infants born by vaginal delivery [44,45]. Assisted vaginal delivery with suction bell or forceps increases the use of supplemental feeding [44]. A recent study that used a prenatal individualised mixed-management intervention to promote breastfeeding reported positive effects on breastfeeding as well as maternal physical and psychological health [46].

The length of the 38 guidelines varied widely, and 16 were limited to one to three pages. More pages can facilitate the inclusion of important information. Three of the 41 clinics did not have any guidelines, which is contrary to The Global Strategy for Infants and Young Child Feeding (WHO) recommendations, stating that infants should not receive supplemental feeding unless it is medically justified [1]. This strengthens the motivation to promote the availability of guidelines for all clinics about when supplemental feeding is medically indicated. There is no reason for clinics to have different guidelines, as all Swedish infants have the right to best practice, evidence-based and equal care. Therefore, we emphasise the importance of having one set of national guidelines for supporting supplemental feeding in Sweden that can be updated as needed.

The authors of 31 guidelines were health professionals; 25 were physicians and 22 midwives. We believe that a multidisciplinary authoring team including midwives, obstetricians, pediatricians and pediatric nurses should be involved in authoring the national guidelines, given their expertise. References were included in 19, and 18 of those referred to the National Care Program—Neonatal hypoglycaemia in infants with gestational age ≥ 35 weeks [2]. It is important to include references to ensure that the content is evidence-based and to enable those interested to read more. The level of evidence GRADE varied in the guideline’s references. We state that guidelines should be evidence-based and include adequate references.

### Methodological Considerations

To ensure that the sample would provide extensive content to address the aim of our study, all women’s clinics in Sweden with associated maternity wards were asked to participate. Hospitals without a maternity ward were excluded because infants with a medical indication for supplemental feeding are usually treated in the maternity ward. Data collection started via mail but, because the response rate was low, reminders were sent both by mail and phone several times, and eventually 41 of 43 (95%) clinics agreed to participate. We speculate that the difficulty we experienced accessing the guidelines was not an unwillingness to share, but rather reflected that the hospitals are overwhelmed with work.

The data were analysed using content analysis with a deductive approach according to Elo and Kyngäs (2008) [31]. We tried to present the results in enough detail to provide a clear understanding and to increase the credibility of our findings. Dependability was achieved because the text was read independently and analysed by researchers with different backgrounds, such as midwifery, midwifery students, paediatric nursing and public health nursing. Transferability was enhanced by analysing nearly all of Sweden’s local guidelines.

## 5. Conclusions and Suggestions for Future Direction

To summarise, most Swedish maternal clinics have guidelines for supplemental feeding, but their content differs. We believe that a set of national guidelines for the supplemental feeding of infants is needed to ensure equality in best practice care, to ensure infant safety and to support parents. Having national guidelines which, hopefully, increase the breastfeeding rate, should benefit both infant and maternal health. We also believe that there should be more national guidelines in general in Sweden because health professionals often move location, and it is easier to update only one set of guidelines. National guidelines can also help to ensure equal care to promote consensus about the quality of care and overall best practices for the population, which is consistent with the Swedish government’s declaration.

## Figures and Tables

**Table 1:** The nine categories.

Category
Author
Year developed
Stated year for next revision
References
Number of pages
Feeding methods
Risk factors
Breastfeeding support
Discontinuation of supplemental feeding

**Table 2:** Descriptive data.

Guidelines *n* = 38			
Component	Included	Omitted	Extra Information
Author n (%)	31 (82)	7 (18)	25 physicians 22 midwifes
Year developed n (%)	34 (89)	4 (11)	2013–2019
Stated year for next revision n (%)	8 (24)	30 (76)	The validity period varied between 2019–2022
References n (%)	19 (50)	19 (50)	18 international, 1 national
Number of pages, mean (min–max)	19 (1–45)		

**Table 3:** Criteria for quality assessment under GRADE based on study design [32].

Grade of Evidence /Level of Recommendation
**High:** Randomised studies. Further research is unlikely to change our confidence in the estimate of effect.
**Moderate:** Further research is likely to have an important impact on our confidence in the estimate of effect and may change the estimate.
**Low:** Observational studies. Further research is very likely to have an important impact on our confidence in the estimate of effect and is likely to change the estimate.
**Very low:** Any estimate of effect is very uncertain.

## Data Availability

The data presented in this study are available in article.
